# LPS-induced CXCR7 expression promotes gastric Cancer proliferation and migration via the TLR4/MD-2 pathway

**DOI:** 10.1186/s13000-019-0780-x

**Published:** 2019-01-12

**Authors:** Nan Li, Huanbai Xu, Yurong Ou, Zhenzhong Feng, Qiong Zhang, Qing Zhu, Zhaogen Cai

**Affiliations:** 1 Department of Pathology, The First Affiliated Hospital of Bengbu Medical College, Bengbu, China; 2 Department of Pathology, Bengbu Medical College, Bengbu, China; 3 Department of Endocrinology and Metabolism, Shanghai Jiaotong University Affiliated First People’s Hospital, Shanghai, China

**Keywords:** Gastric cancer, LPS, CXCR7, TLR4, MD-2

## Abstract

**Background:**

Lipopolysaccharide (LPS) from *Helicobacter pylori (HP)* plays an important role in gastric cancer occurrence and development. Toll-like receptor 4 (TLR4) and myeloid differential protein-2 (MD-2) are also reported to be involved in gastric cancer cell proliferation and invasion. CXC chemokine receptor 7 (CXCR7), a second receptor for CXCL12, has been detected in multiple types of tumor tissues. Nevertheless, the biological function and regulation of CXCR7 and its relationship with TLR4 and MD-2 in gastric cancer are not completely understood and therefore warrant further study.

**Methods:**

CXCR7 expression was examined in 150 gastric cancer tissues using immunohistochemistry (IHC). RT-PCR and western blotting were used to detect CXCR7 expression in several gastric cancer cell lines (SGC7901, AGS, MGC-803, MKN-45 and BGC823). shRNAs were designed using a pGPU6/GFP/Neo vector. A CCK-8 assay was used to assess cell proliferation, and transwell assays were performed to assess cell migration. In addition, a gastric cancer xenograft model was generated.

**Results:**

The LPS-TLR4-MD-2 pathway elevates CXCR7 expression in SGC7901 cells, and TLR4/MD-2-mediated increases in CXCR7 levels modulate the proliferation and migration of tumor cells. Knockdown of TLR4 and MD-2 demonstrated that both are essential for LPS-induced CXCR7 expression, which in turn is responsible for LPS-induced SGC7901 cell proliferation and migration. Moreover, higher TLR4, MD-2 and CXCR7 expression was detected in gastric cancer tissues than in paracancerous normal control tissues. The expression levels of TLR4, MD-2 and CXCR7 were closely related to gastric cancer TNM stage and lymph node metastasis. In an animal model, significant differences in CXCR7 expression in tumor masses were observed between the control group and experimental group.

**Conclusions:**

The results of this study indicate that CXCR7 plays an important role in gastric cancer progression via inflammatory mechanisms, suggesting that CXCR7 could provide a basis for the development and clinical application of a targeted drug for gastric cancer.

## Background

Gastric cancer is one of the most common malignant tumors and is the third leading cause of cancer mortality worldwide [1]. Chronic inflammation is correlated with many malignant tumors [2, 3], and chronic atrophic gastritis with intestinal metaplasia is associated with an increased incidence of gastric cancer compared with the normal population [4]. *Helicobacter pylori (HP)* is considered a primary carcinogen in gastric cancer pathogenesis by the WHO. Consequently, chronic inflammation induced by *HP* plays an important role in gastric cancer occurrence and development [3, 5–8]. Lipopolysaccharide (LPS) is a toxic component of the outermost layer of the *HP* cytoderm and may contribute to long-term inflammatory injury in the gastric mucosa [9, 10].

Transmembrane Toll-like receptors (TLRs) are a class of signal transduction proteins referred to as pattern recognition receptors that can specifically recognize pathogen-related molecular patterns (PAMPs). TLR4 was first discovered as a human TLR that is sensitized by LPS from Gram-negative bacteria [11, 12]. Related research found that TLR4 is upregulated in gastric cancer tissues and has low expression in normal gastric mucosa [13, 14]. LPS can induce the formation of an LPS-TLR4-MD-2 multimer by complexing with TLR4 and myeloid differential protein-2 (MD-2), which can further activate proinflammatory signaling pathways and facilitate the expression of corresponding cytokines and receptors [15, 16]. Consequently, the pathogenic mechanism of TLR4 in gastric cancer progression should be studied. CXC chemokine receptor 7 (CXCR7), a second receptor for CXCL12, has been detected on the surface of multiple types of tumor tissues [17, 18]. Nevertheless, the biological function and regulation of CXCR7 and its relationship with TLR4 and MD-2 in gastric cancer are still not completely understood and are therefore worthy of study.

In the present study, we explored the function of CXCR7 in the LPS/TLR4/MD-2 regulatory pathway in gastric cancer. LPS exposure increased the expression of CXCR7 in gastric cancer SGC7901 cells, which highly express TLR4/MD-2. Moreover, LPS stimulation induced CXCR7 expression in gastric cancer via TLR4/MD-2 signaling to promote the proliferation and migration of SGC7901 cells. Furthermore, tumor-bearing nude mice and clinicopathology were used to verify that the LPS/TLR4/CXCR7 pathway may be critical during gastric cancer development. These results suggest that the connection between inflammation and gastric cancer may enable the development of novel methods for inhibiting tumor occurrence and development.

## Methods

### Tissue specimens

From 2012 to 2016, tissues from one hundred fifty gastric cancer patients (all gastric adenocarcinoma) were obtained from the Department of Pathology, The First Affiliated Hospital of Bengbu Medical College. Paracancerous normal tissues from sixty patients were used as controls. Histopathologic examination was performed by two pathologists to verify the histological diagnosis. Tumor staging was determined according to the Union for International Cancer Control (UICC) and the American Joint Committee on Cancer (AJCC) gastric cancer TNM staging system (8th edition).

### Cell lines and main reagents

The gastric cancer cell lines SGC7901, AGS, MGC-803, MKN-45, BGC823 and GES-1 were purchased from the Type Culture Collection of Chinese Academy of Sciences (Shanghai, China). LPS-EB (LPS from *E. coli* O111:B4, cat. no. tlrl-eblps) was purchased from InvivoGen, USA. TLR4 monoclonal antibody (clone ab22048) and an MD-2 monoclonal antibody (clone ab24182) were obtained from Abcam, England. CXCR7 monoclonal antibody (clone MAB4227) and CXCL12 antibody (cat. no. 2716-SD-025/CF) were obtained from R&D systems, USA. CXCR7 antagonist (CCX771) was kindly provided by ChemoCentryx. CXCR4 antagonist (AMD3100) was obtained from Adooq Bioscience, USA. HRP-conjugated secondary antibodies for western blotting (cat. no. ab6734) were purchased from Abcam.

### Cell cultivation and transfection

AGS cells were cultured in F12 medium (cat. no. 12-615F, Lonza, CH) supplemented with 20% fetal bovine serum (FBS), penicillin (100 U/ml), and streptomycin (100 mg/ml) at 37 °C in 5% CO_2_. The other cells were grown in RPMI-1640 medium (cat. no. 11875093, Gibco, USA) supplemented with 10% FBS, penicillin (100 U/ml), and streptomycin (100 mg/ml) at 37 °C in 5% CO_2_.

Specific siRNA sequences against TLR4 (5’-GAGCCGCUGGUGUAUCUUU-3′) and MD-2 (5’-GUGGGAGAGAUUUAAAGCA-3′), as well as a scrambled control siRNA (5’-AGGACTGAGTGTACCGTCT-3′ (Scram)), were designed as shRNAs and inserted into the pGPU6/GFP/Neo vector (GenePharma, Shanghai, China) under the control of a U6 promoter. Cells resistant to G418 (800 μg/mL) were selected and cultivated for further study [19, 20]. The depletion of endogenous TLR4 or MD-2 by shRNA treatment was determined by RT-PCR and western blotting analyses (performed by GenePharma Biotechnology Company in Shanghai). Cell transfections were performed with Lipofectamine 2000 (cat. no. 11668-027, Invitrogen, USA) based on the manufacturer’s protocol.

### Reverse transcription PCR and quantitative real-time PCR

The upstream primers and downstream primers for TLR4, MD-2, CXCR7 and GAPDH are shown in Table 1 [15]. Total RNA was extracted from cells or tissues with TriPure Isolation Reagent (Roche, cat. no. 11667165001) according to the standard protocol. A NanoDrop® 8000 spectrophotometer (Thermo Scientific) was used to determine the total RNA concentration. cDNA was obtained through reverse transcription of 2 μg of total RNA in a 10 μl reaction. PCR was performed according to the instructions. The expression products were electrophoresed on a 1% agarose gel and photographed. Quantitative real-time RT-PCR (qRT-PCR) was performed with template cDNA using SYBR Premix Ex TaqII (Tli RNaseH Plus; Takara). An ABI PRISM 7900HT sequence detection system was used to detect the mRNA levels of the target genes, which were normalized to the levels of GAPDH.

**Table 1:** The upstream primers and downstream primers for TLR4, MD-2, CXCR7 and GAPDH

Gene	Primer	Sequence (5′ → 3′)
TLR4	Forward	TGCAATGGATCAAGGACCAGAGG
	Reverse	TGCAGCCAGCAAGAAGCATCAG
MD-2	Forward	CCGAGGATCTGATGACGATTA
	Reverse	GGCTCCCAGAAATAGCTTCAA
CXCR7	Forward	CACAGCACAGCCAGGAAGG
	Reverse	GTTCCCTGGCTCTGAGTAGTCGA
GAPDH	Forward	GGATTTGGTCGTATTGGG
	Reverse	GGAAGATGGTGATGGGATT

parameter cases TLR4^+^ *P* value MD-2^+^ *P* value CXCR7^+^ *P* value

### Western blot analysis

Gastric cancer SGC7901 cells were lysed in RIPA lysis buffer (USBiological, USA) supplemented with a protease inhibitor cocktail (cat. no. B14001, Biotool, USA). The total protein level in the nuclear lysates was measured using the Pierce™ BCA Protein Assay Kit (Thermo Fisher Scientific). Protein lysates (60 μg) were separated on 10% SDS-PAGE gels. The proteins were transferred to nitrocellulose using an iBlot 2 Dry Blotting System (Thermo Scientific, USA). The membranes were blocked with 5% dry skim milk in PBST at room temperature for 2 h. Blots were probed with mouse monoclonal primary antibodies against MD-2, TLR4 and CXCR7 and then incubated with the appropriate HRP-conjugated secondary antibodies. The membranes were washed, and the immunoreactive bands were observed using a West Pico chemiluminescence system (Pierce). The protein expression level was analyzed compared with the GAPDH level.

### Cell proliferation assay

Gastric cancer SGC7901 cells were seeded in 96-well plates (2 × 10^3^ cells per well) in 100 μl of medium containing 1% FBS with or without LPS (500 ng/ml) and cultured for 12, 24 and 48 h. In certain experiments, SGC7901 cells were pretreated (1 h) with CCX771 (1 μM). After 48 h, a CCK-8 assay (Abbkine, USA) was used to assess cell proliferation. Each experiment was sampled in duplicate, and the data are presented as the mean ± SD of three independent experiments.

### Cell migration assay

A transwell assay (Chemicon) was performed to assess cell migration. Gastric cancer SGC7901 cells were resuspended in 1% FBS-medium (5 × 10^5^ cells/ml) and seeded into the upper transwell chambers containing 0.5% BSA. Then, 1% FBS-medium without (control) or with CXCL12 (100 ng/ml) was added to the lower chambers. In certain experiments, SGC7901 cells were pretreated (1 h) with CCX771 (1 μM). The experiments were performed at 37 **°**C and 5% CO_2_ for 24 h. After incubation, the nonmigrated cells on the upper surface of the filters were removed, and the hematoxylin-stained migrated cells on the lower surface were counted in five fields under a microscope. For quantification, each experiment was performed in triplicate.

### Immunohistochemistry (IHC) staining and scoring

All archival paraffin blocks were numbered separately and cut into serial 4-μm-thick sections and subjected to routine deparaffinization and rehydration. Antigen retrieval, inhibition of endogenous peroxidase activity and blocking of nonspecific binding were performed according to the kit instructions. The primary antibodies used were anti-TLR4 (mouse monoclonal antibody, Abcam, US), anti-MD-2 (rabbit polyclonal antibody, Abcam, US) and anti-CXCR7 (rabbit polyclonal, Boster Biological Technology, Wuhan, China). Rat anti-mouse IgG2b-HRP (Catalog No. SBA-1186-05, SouthernBiotech, USA) was used as a second antibody. Positive tissue slices were used as positive controls, and phosphate-buffered saline (PBS) was used instead of a primary antibody as a negative control.

The IHC staining was scored according to the percentage of positive tumor cells and the intensity of the staining. The percentage of positive tumor cells was divided into four grades: 0 (no staining was observed), 1 (< 10% of cells were positively stained), 2 (10–50% of cells were positively stained), and 3 (> 50% of cells were positively stained). The staining intensity was scored as 0 (no staining or faint yellow), 1 (light yellow), 2 (brown), and 3 (dark brown). The final IHC score was obtained by multiplying the staining intensity score by the percentage of positive tumor cells. Scores of 0–2 were negative, and those of 3 to 9 were positive.

### Animals and tumor model

Four- to six-week-old athymic nude mice (BALB/C-nu/nu) were purchased from the Shanghai Laboratory Animal Center at the Chinese Academy of Sciences and raised in a specific-pathogen-free facility at Bengbu Medical College. All of the animal procedures were approved by the Animal Welfare & Ethics Committee of Bengbu Medical College. The tumors were xenografted into the left flank of nude mice through subcutaneous injection of 2 × 10^6^ SGC7901 cells in 50 μl of PBS. The mice were intratumorally injected with LPS (400 μg/kg) or the same volume of DMSO every other day. The tumor growth in the different groups was observed every 4 days, and the tumor volume was measured with calipers and calculated using the following formula: larger diameter×(smaller diameter)^2^/2. When the tumor volume reached approximately 1 cm^3^, the mice were euthanized. The tumor tissues were removed, and CXCR7 expression was analyzed via IHC.

### Statistical analysis

The data were analyzed using the Statistical Package for Social Science software (version 19.0, SPSS Inc., Chicago, IL). The qualitative data for comparisons between groups were assessed with a chi square test and two-sided Fisher’s exact test. All quantitative data are presented as the mean ± SD of three independent experiments. Student’s t-test was applied to assess differences between groups. A *P* value less than 0.05 was considered statistically significant.

## Results

### Expression of TLR4, MD-2 and CXCR7 in gastric cancer cell lines

**Fig. 1:**
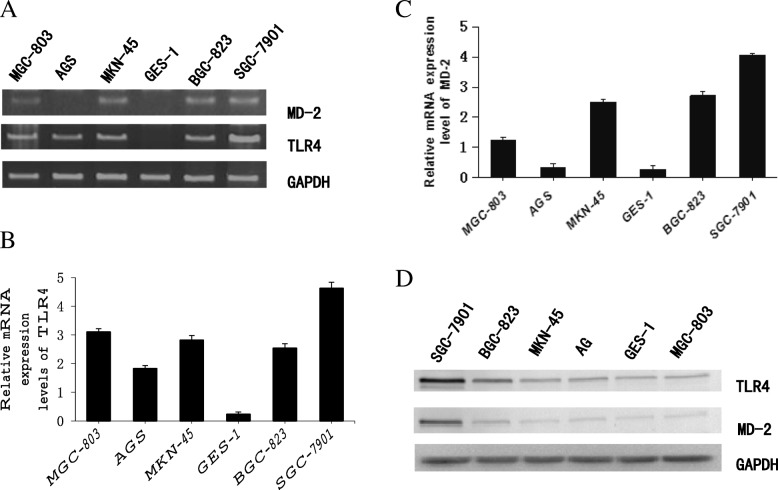
TLR4 and MD-2 expression in gastric cancer cell lines. The expression of TLR4 and MD-2 was analyzed via RT-PCR (**a**), qRT-PCR (**b, c**) and western blotting (**d**) in gastric cancer cell lines

We investigated the mRNA and protein expression levels of TLR4 and MD-2 in human gastric cancer cell lines (SGC-7901, AGS, MGC-803, BGC-823 and MKN-45) and normal gastric epithelial GES-1 cells using RT-PCR and western blotting. The results showed that the mRNA and protein expression of both TLR4 and MD-2 was higher in gastric cancer lines than in normal gastric epithelial GES-1 cells (Fig. 1a-d). The highest expression level of both TLR4 and MD-2 was observed in the SGC-7901 cell line. Additionally, we detected CXCR7 expression in the above cell lines and found that CXCR7 expression was low in general. By comparison, the level of CXCR7 expression was slightly higher in SGC7901 cells, whereas CXCR4 expression was relatively high in these cells. Accordingly, the SGC7901 cell line was selected to study the TLR4/MD-2 signaling pathway.

### LPS elevates CXCR7 expression to promote SGC7901 cell proliferation and migration

**Fig. 2:**
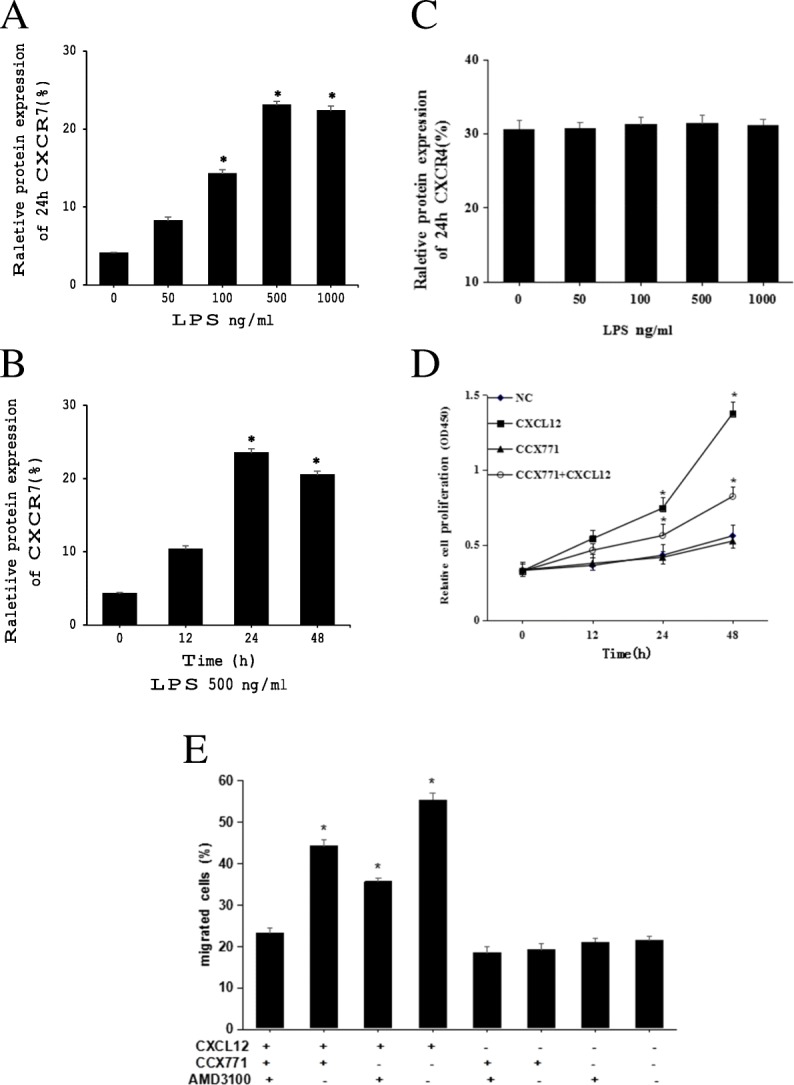
LPS induced changes in CXCR7 expression in SGC7901 cells. A: CXCR7 protein expression was assessed by western blotting after SGC7901 cells were stimulated for different periods of time with 500 ng/mL LPS; B: SGC7901 cells were cultured with various concentrations of LPS for 24 h, and CXCR7 protein expression was analyzed via western blotting; C: After exposure of SGC7901 cells to LPS (500 ng/ml), CXCR4 protein expression was assessed by western blotting; D and E: After pretreatment with CCX771, SGC7901 cell proliferation and migration were largely inhibited in response to CXCL12 (100 ng/ml) after 48 h of incubation with LPS. *, *P* < 0.05, vs. the NC group. The data are presented as the mean ± SD

We treated SGC7901 cells with LPS and observed the effects on proliferation and migration through CXCR7. When SGC7901 cells were stimulated with different concentrations of LPS, CXCR7 expression increased in a concentration-dependent manner up to 500 ng/mL (Fig. 2a). When cells were treated with 500 ng/mL of LPS for 12, 24 and 48 h, CXCR7 expression increased in a time-dependent manner up to 24 h (Fig. 2b). In contrast with LPS-induced CXCR7 expression changes in SGC7901 cells, LPS-induced CXCR4 expression did not exhibit a marked increase (Fig. 2c). We next used CCX771 (a new CXCR7-specific antagonist) and CXCL12 (an endogenous CXCR7 ligand) to examine the role of CXCR7 in regulating SGC7901 cell proliferation and migration using CCK8 and transwell assays. Because CXCR4 is another receptor for CXCL12, SGC7901 cells were pretreated with AMD3100 (a CXCR4 antagonist) in some of the experiments. Upon CXCL12 stimulation and incubation with LPS, we found that SGC7901 cells exhibited significant proliferation and migration after 48 h. After SGC7901 cells were pretreated with CCX771 or AMD3100, the proliferation- and migration-stimulating effects were reduced in the presence of CXCL12. However, CXCR7 promoted proliferation and migration more obviously than CXCR4 (Fig. 2d-e).

### LPS-induced CXCR7 expression is blocked by knockdown of TLR4 and MD-2

**Fig. 3:**
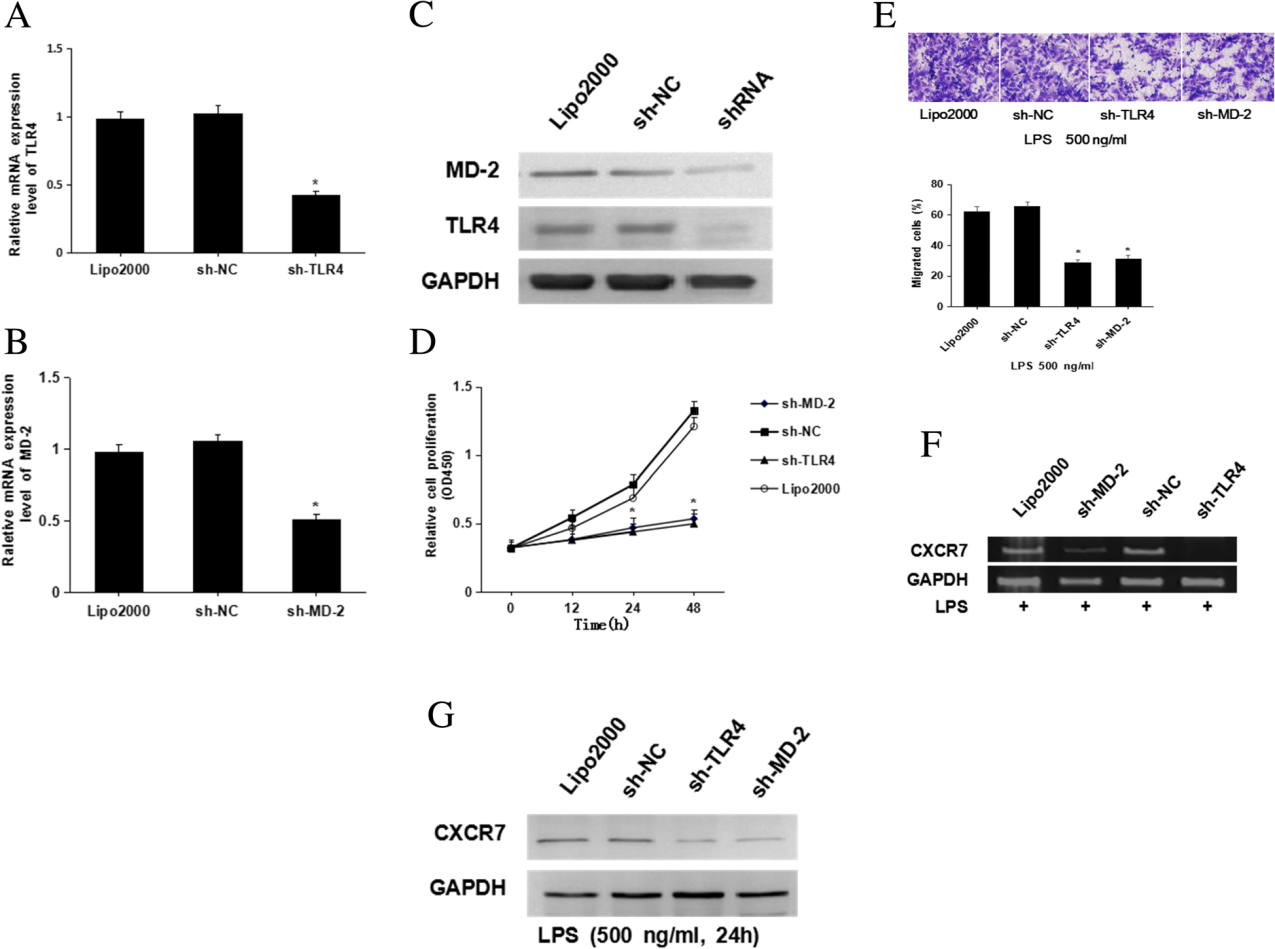
The effect of TLR4 and MD-2 knockdown on LPS-induced CXCR7 expression. **a**-**c**: Gastric cancer cells were transfected with TLR4-specific or MD-2-specific shRNAs, and endogenous TLR4 and MD-2 expression levels were analyzed via qRT-PCR (**a** and **b**) and western blotting (**c**); **d** and **e**: Gastric cancer cells were transfected with TLR4-specific or MD-2-specific shRNAs and treated with 500 ng/mL LPS. A CCK-8 assay was then used to detect cell proliferation (**d**), and a transwell assay was used to assess cell migration (**e**); **f** and **g**: As described in D and E, CXCR7 expression was analyzed via RT-PCR (F) and western blotting (**g**) *, *P* < 0.05, vs. the Lipo2000 group and sh-NC group. The data are presented as the mean ± SD

We next investigated whether LPS-induced CXCR7 expression promotes SGC7901 cell proliferation and migration via the TLR4/MD-2 pathway. SGC7901 cells were transfected with specific shRNAs targeting TLR4 and MD-2. When endogenous TLR4 and MD-2 were knocked down by shRNA (Fig. 3a-c), LPS-induced SGC7901 cell proliferation and migration were decreased compared with the scrambled and negative control groups (Fig. 3d-e). Moreover, after SGC7901 cells transfected with anti-TLR4 and anti-MD-2 shRNA were coincubated with LPS, CXCR7 mRNA and protein expression levels did not increase. By comparison, CXCR7 expression was markedly decreased in SGC7901 cells transfected with anti-TLR4 and anti-MD-2 shRNAs compared with the control group (Fig. 3f-g). In general, these results suggest that the TLR4/MD-2 signaling pathway is essential for the LPS-induced increase in CXCR7 expression and its promotion of SGC7901 cell proliferation and migration.

### IHC results for TLR4, MD-2 and CXCR7 and their relationship with clinicopathologic factors

**Fig. 4:**
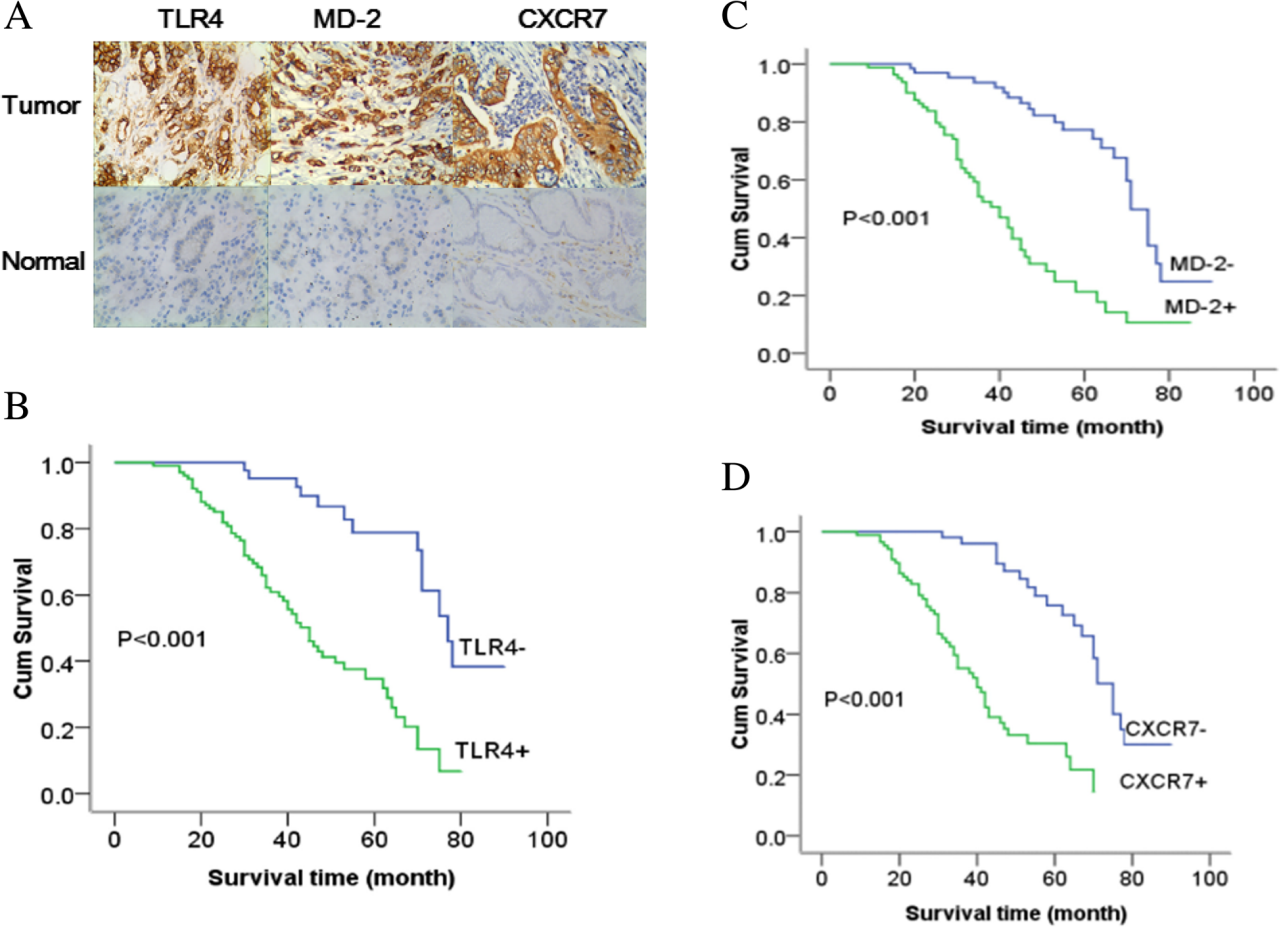
TLR4, MD-2 and CXCR7 expression in gastric cancer indicates poor prognosis. **a**: Representative immunohistochemical staining of TLR4, MD-2 and CXCR7 in gastric cancer tissues and paracancerous tissues (original magnification 400×); survival curve for patients with gastric cancer expressing TLR4 (**b**), MD-2 (**c**) and CXCR7 (**d**)

We used IHC to analyze the clinicopathologic relevance of TLR4, MD-2 and CXCR7 expression in gastric cancer tissues. The expression rate of TLR4, MD-2 and CXCR7 in gastric cancer tissues was 68% (102/150), 54% (81/150) and 59.3% (89/150), respectively, and these rates were significantly different from those in normal gastric tissues (Fig. 4a). Our data revealed significant relationships between high TLR4, MD-2 and CXCR7 expression and lymph node metastasis and TNM stage (Table 2). However, the expression of these proteins was not significantly correlated with gender, age, tumor diameter or differentiation degree, although the expression of CXCR7 was slightly related to tumor diameter. We also investigated potential correlations between the protein expression of CXCR7 and that of TLR4 and MD-2, and we found the relationships to be significant (Table 3). In addition, the survival analysis demonstrated that the median overall survival time among 150 patients was 58.0 months. The overall survival of TLR4-positive patients was 45.0 months (95% CI, 39.2–50.8), whereas the rate was 77.0 months (95% CI, 69.6–84.4) for TLR4-negative patients (Fig. 4b). The overall survival for MD-2- and CXCR7-positive patients (40.0 months and 40.0 months, respectively [95% CI, 33.9–46.1, 33.7–46.3]) was lower than that for MD-2- and CXCR7-negative patients (71.0 months and 75.0 months, respectively [95% CI, 66.8–75.2, 69.8–80.2]) (Fig. 4c-d).

**Table 2:** Relationship between TLR4, MD-2, and CXCR7 protein expression and clinicopathological parameters in gastric cancer

Gender							
Male	90	63	0.520	32	0.894	52	0.635
Female	60	39		49		37	
Age (yrs)							
< 60	79	51	0.340	36	0.443	45	0.533
≥60	71	51		45		44	
Diameter (cm)							
< 5.0	93	67	0.175	53	0.348	61	0.046
≥5.0	57	35		28		28	
Lauren type							
Intestinal type	71	50	0.192	35	0.273	44	0.533
Diffuse type	79	52		46		45	
Differentiation							
Well-moderately	92	59	0.201	51	0.657	53	0.588
Poorly	58	43		30		36	
Lymph node metastasis							
+	60	47	0.027	42	0.001	42	0.030
–	90	55		39		47	
TNM stage							
I + II	83	47	0.001	38	0.025	37	< 0.001
III + IV	67	55		43		52

**Table 3:** The correlation between the expression of CXCR7 and TLR4 and MD-2 in gastric carcinoma

CXCR7	n	TLR4		MD-2	
		(+)	(%)	(+)	(%)
Positive	89	82	(92.1)	60	(67.4)
Negative	61	20	(32.8)	21	(33.3)
χ^2^		58.6		15.9	
P value		< 0.001		< 0.001	

### LPS stimulation of SGC7901 cells promotes tumor growth in nude mice

**Fig. 5:**
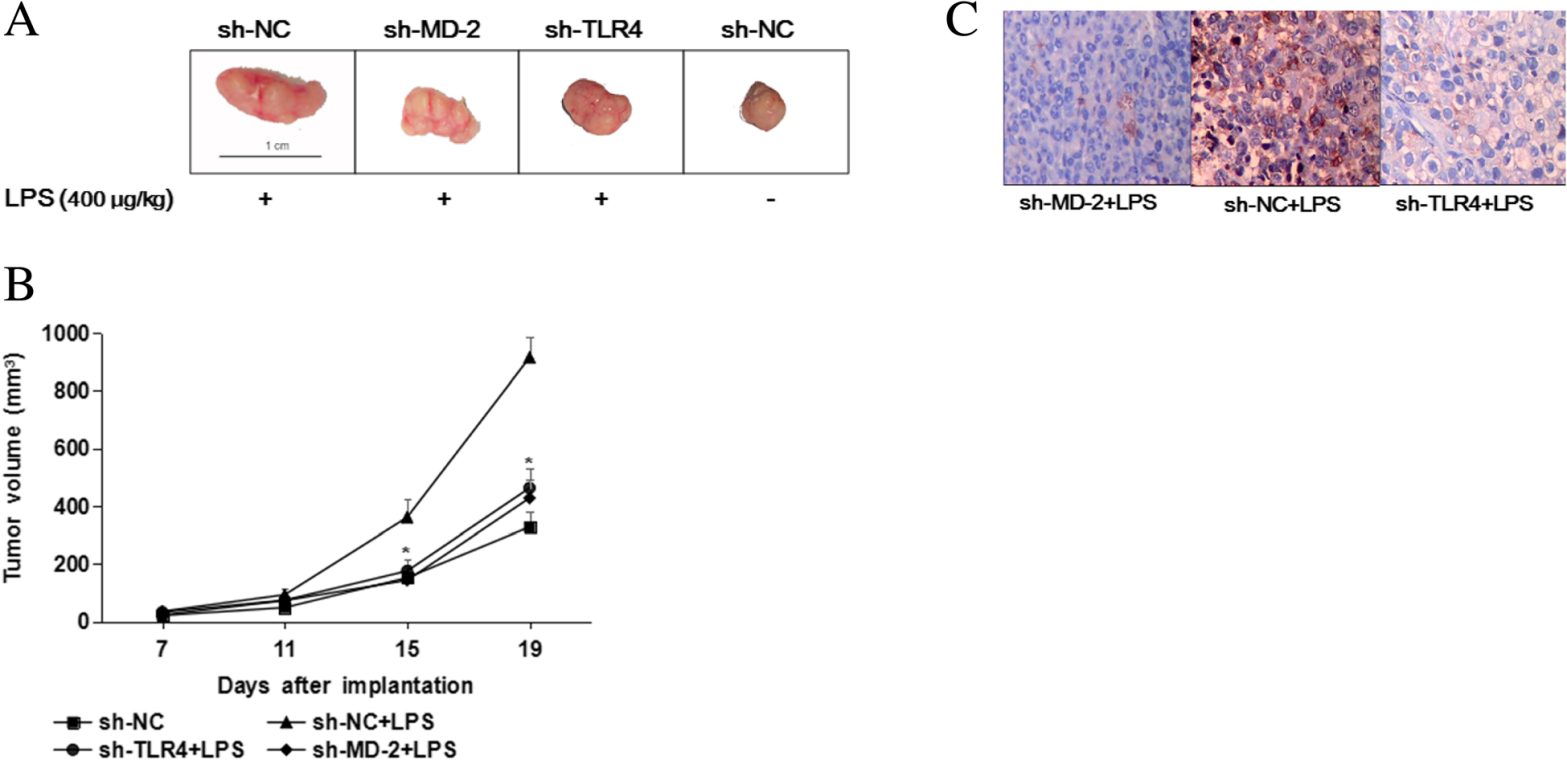
LPS enhances the tumorigenicity of SGC7901 cells via the TLR4/MD-2 pathway. **a** and **b**: SGC7901 cells (2 × 10^6^ cells/mice) treated with different shRNAs (sh-NC, sh-TLR4 and sh-MD-2) were injected subcutaneously into the flanks of nude mice, and the mice were intratumorally injected with LPS (400 μg/kg) every other day. The tumor volume was observed (**a**) and measured (**b**); *, *P* < 0.05. The data are presented as the mean ± SD; C: CXCR7 expression in tumors was analyzed via immunohistochemistry (original magnification 400×)

To assess the effect of the TLR4/MD-2 pathway on tumorigenicity in vivo, we established a nude mouse xenograft model. The flanks of athymic nude mice were subcutaneously injected with SGC7901 cells with or without LPS treatment. The tumor bulk and mass in the LPS treatment groups were increased compared with those in the control groups. When TLR4 or MD-2 was inhibited using shRNA in SGC7901 cells, LPS-induced tumor growth was considerably restrained in the knockdown groups compared with the scrambled shRNA group (Fig. 5a, b). Additionally, the expression of CXCR7 in the TLR4 and MD-2 knockdown groups was reduced compared with that in the scrambled shRNA group (Fig. 5c, Table 4). These results indicate that LPS-induced CXCR7 expression may enhance gastric cancer tumor growth in vivo through the TLR4/MD-2 pathway.

**Table 4:** The expression of CXCR7 protein in xenograft cells

group	n	expression of CXCR7	
		(−)	(+)
sh-TLR4 + LPS	6	5*	1
sh-MD-2 + LPS	6	5*	1
sh-NC + LPS	6	1	5
sh-NC	6	2	4

^*^vs. sh-NC + LPS and sh-NC group, *P* < 0.05

## Discussion

Gastric cancer is one of the most common malignant tumors worldwide. *HP* infection is involved in gastric carcinogenesis and gastric cancer development. LPS is a lipid and polysaccharide complex that is a unique cell wall component in Gram-negative bacteria [19]. *HP* LPS is also the primary endotoxin and has a structure and characteristics similar to those of *Escherichia coli* LPS (*E. coli*-LPS). Additionally, LPS is the main virulence factor of *HP* and is released from damaged cells or bacteria in tumor tissues [20]. Numerous studies have addressed the key mechanism by which inflammation-induced LPS signaling alters the invasive and metastatic potential of gastric cancer cells. In the present study, we confirmed that LPS can induce and promote SGC7901 cell proliferation and metastasis.

TLR4 activation can promote tumor progression by promoting apoptosis resistance, invasion, metastasis, and immune surveillance evasion. TLR4 is upregulated in many solid tumors, including gastric cancer [14, 21, 22]. LPS binds to TLR4 in tumor tissues and induces the synthesis of a variety of inflammatory mediators, including TNFa, IL8 and multiple chemokines. Subsequently, an inflammatory microenvironment is generated via MyD88-dependent and independent pathways, promoting tumor development [23–25]. Relevant clinical and experimental studies have shown that CXC chemokines, such as the CXCL12 (SDF-1)/CXCR4 axis, can promote tumor growth, invasion and angiogenesis [26–29]. CXCR7 was originally termed RDC-1 (an orphan receptor). Recently, CXCR7 has been shown to cause chemotaxis in T lymphocytes in response to CXCL12 (the CXCR4 ligand) and was thus identified as a second CXCL12 receptor. Growing evidence suggests that CXCR7 can activate downstream signal transduction molecules, impact cell adhesion and invasion and further promote tumor cell proliferation through a complex signaling cascade [30–33]. Reports on the relationship between CXCR7 and gastric cancer are relatively rare, and the exact mechanism underlying how CXCR7 promotes the occurrence and development of gastric cancer requires further study. In this study, our data elucidated the key mechanisms underlying the promotive effect of inflammation-derived LPS signaling on the metastatic potential of SGC7901 cells. LPS can upregulate CXCR7 expression through TLR4 signaling, thereby promoting gastric cancer cell proliferation and migration. Moreover, higher TLR4 and CXCR7 expression levels were found in gastric cancer tissues than in paracancerous normal tissues. TLR4 knockdown in SGC7901 cells revealed that this receptor is essential for LPS-induced CXCR7 expression. Additionally, we established a nude mouse xenograft model to further assess the function of the LPS/TLR4/CXCR7 pathway in gastric cancer. The results indicated that TLR4 and CXCR7 expression is related to gastric cancer growth and metastatic potential.

MD-2 is a recently discovered secreted glycoprotein. As an important regulator of innate immune recognition processes, MD-2 is a receptor molecule necessary for LPS to activate the TLR4 transmembrane signaling pathway [34, 35]. Among all of the TLR4 accessory molecules, MD-2 is essential for the response to LPS [36, 37]. MD-2 mediates the release of proinflammatory cytokines through the formation of TLR4/MD-2 complexes following the recognition of bacterial LPSs. However, the regulatory mechanism and relationship between TLR4-MD-2 and CXCR7 in promoting gastric cancer development remains unknown. In the present study, we aimed to elucidate the connection between TLR4-MD-2 and CXCR7. The results indicated that CXCR7 expression was significantly increased in TLR4/MD-2-positive LPS-stimulated SGC7901 cells. Notably, LPS-mediated CXCR7 expression was blocked by TLR4 or MD-2 knockdown. Moreover, LPS did not affect CXCR7 expression in SGC7901 cells that expressed TLR4 but not MD-2. This result supports the notion that MD-2 is essential to the TLR4 signaling pathway. In addition, the expression level of MD-2 was significantly higher in gastric cancer tissues than in paracancerous control tissues. MD-2 expression was highly correlated with lymph node metastasis and TNM stage. Furthermore, our analysis of gastric cancer tissues from 150 patients showed that CXCR7 expression is positively correlated with that of TLR4 and MD-2 and that these three factors predict a worse prognosis and poorer survival. Nevertheless, more evidence should be obtained to confirm the mechanism underlying TLR4/MD-2 signaling through CXCR7 and the synergistic action of these molecules in tumor development and progression.

## Conclusions

In summary, the LPS/TLR4 pathway is linked to gastric cancer development, and the interaction between TLR4, MD-2 and CXCR7 is closely related to tumor growth and metastasis. Understanding the relationship between microbiological signals and gastric cancer could have a major impact on tumor prevention and treatment.

## Abbreviations

CXCR7: CXC chemokine receptor 7
*HP*: *Helicobacter pylori*
IHC: Immunohistochemistry
LPS: Lipopolysaccharide
MD-2: Myeloid differential protein-2
PAMPs: Pathogen-related molecular patterns
RT-PCR: Real-time polymerase chain reaction
TLR4: Toll-like receptor 4
